# HLA and proteasome expression body map

**DOI:** 10.1186/s12920-018-0354-x

**Published:** 2018-03-27

**Authors:** Sebastian Boegel, Martin Löwer, Thomas Bukur, Patrick Sorn, John C. Castle, Ugur Sahin

**Affiliations:** 1 TRON gGmbH - Translational Oncology at Johannes Gutenberg, University Medical Center gGmbH, Freiligrathstr 12, Mainz, Germany; 2 Present address: Agenus Inc, Lexington MA, 02421 USA

**Keywords:** Human leukocyte antigens, Proteasome, Atlas, Bioinformatics, Autoimmune, HLA expression, Immunology, RNA-Seq, NGS

## Abstract

**Background:**

The presentation of HLA peptide complexes to T cells is a highly regulated and tissue specific process involving multiple transcriptionally controlled cellular components. The extensive polymorphism of HLA genes and the complex composition of the proteasome make it difficult to map their expression profiles across tissues.

**Methods:**

Here we applied a tailored gene quantification pipeline to 4323 publicly available RNA-Seq datasets representing 55 normal tissues and cell types to examine expression profiles of (classical and non-classical) HLA class I, class II and proteasomal genes.

**Results:**

We generated the first comprehensive expression atlas of antigen presenting-related genes across 56 normal tissues and cell types, including immune cells, pancreatic islets, platelets and hematopoietic stem cells. We found a surprisingly heterogeneous HLA expression pattern with up to 100-fold difference in intra-tissue median HLA abundances. Cells of the immune system and lymphatic organs expressed the highest levels of classical HLA class I (HLA-A,-B,-C), class II (HLA-DQA1,-DQB1,-DPA1,-DPB1,-DRA,-DRB1) and non-classical HLA class I (HLA-E,-F) molecules, whereas retina, brain, muscle, megakaryocytes and erythroblasts showed the lowest abundance. In contrast, we identified a distinct and highly tissue-restricted expression pattern of the non-classical class I gene HLA-G in placenta, pancreatic islets, pituitary gland and testis. While the constitutive proteasome showed relatively constant expression across all tissues, we found the immunoproteasome to be enriched in lymphatic organs and almost absent in immune privileged tissues.

**Conclusions:**

Here, we not only provide a reference catalog of tissue and cell type specific HLA expression, but also highlight extremely variable expression of the basic components of antigen processing and presentation in different cell types. Our findings indicate that low expression of classical HLA class I molecules together with lack of immunoproteasome components as well as upregulation of HLA-G may be of key relevance to maintain tolerance in immune privileged tissues.

**Electronic supplementary material:**

The online version of this article (10.1186/s12920-018-0354-x) contains supplementary material, which is available to authorized users.

## Background

One of the key structural elements of the mammalian adaptive immune system is the Major Histocompatibility Complex (MHC). A primary task of the classical MHC molecules is to bind and present self, abnormal self (e.g., neo-epitopes arising from mutations [1]) and foreign (e.g., pathogenic) peptide antigens derived from intracellular (class I) or from extracellular proteins (class II) on the surface of the cell, where the peptide-MHC complex can be screened by T lymphocytes, eventually leading to an immune response. The human MHC system, Human Leukocyte Antigen (HLA), is located on chromosome six and consists of highly polymorphic HLA class I and II genes. As these are co-dominantly expressed, every individual carries two alleles of the HLA class I and class II genes.

There is increasing evidence that diseased cells down-regulate or even loose classical HLA class I and class II expression to escape T-cell mediated killing and non-classical HLA class I molecules have been associated to play a role in cancer immunosuppression [2]. In addition, dysregulation of components of the antigen processing and presenting machinery (APM) in cancer results in alterations of HLA expression, which might result in decreased immunogenicity and lack of T cell recognition [3]. A crucial component of the APM is the proteasome, which is a multi-enzyme complex in the cytosol constantly degrading proteins and thus producing peptide fragments that are transported into the endoplasmatic reticulum by transporter proteins (TAP1, TAP2), where they are further trimmed, loaded onto a HLA class I molecules and transported to the cell surface via the Golgi apparatus [4]. While PSMB5, PSMB6, PSMB7 are ubiquitously expressed components of the constitutive proteasome, the immunoproteasome is generated by overexpression of PSMB8 (LMP7), PSMB9 (LMP2) and PSMB10 (LMP10) genes upon stimulation with INFγ or TNFα. It produces a distinct pool of HLA class I epitopes that are more efficient in activating cytotoxic T cells.

Non-classical HLA class I molecules (HLA-E, -F, −G) have a similar protein structure to that of the classical HLA class I alleles and also require a bound peptide in the binding groove to form a stable complex. However, they are characterized by few allelic polymorphisms and play a role in regulating innate immune responses. Further, they have expression patterns often related to their function, such as HLA-G expression in the placenta where it interacts with maternal effector cells [5].

Understanding HLA and proteasome expression in physiological state is crucial for biomarker discovery, for the development of cancer vaccines targeting neo-epitopes, and for understanding immune escape mechanisms in cancer. Measuring HLA expression levels is often performed via Fluorescence-activated Cell Sorting (FACS), Immunohistochemistry (IHC) or Western Blot using antibodies recognizing HLA proteins and via real-time quantitative polymerase chain reaction (RT-qPCR) using specific primers for HLA mRNA. In contrast, next-generation sequencing (NGS) RNA-Seq [6], recovers millions of nucleotide sequence reads derived from a sample’s transcriptome. Standard RNA-Seq processing algorithms map these reads to a reference genome to reconstruct the transcriptome and derive the abundance of the genes. However, the reference does not reflect the polymorphic nature of the HLA system, precluding accurate HLA typing [7] and thus expression determination. To account for this, we developed the algorithm ‘seq2HLA’ [8] that takes bulk RNA-Seq raw data as input and outputs the most probable 4-digit HLA class I and II types [9] and locus-specific expression.

Here, we extended seq2HLA to determine the HLA type and expression of non-classical HLA class I molecules. Using the updated algorithm, we analyzed 4323 public RNA-Seq datasets to generate – to our knowledge - the first comprehensive expression atlas of HLA class I (classical and non -classical) and class II gene loci across 38 non-cancer tissues and 17 different cell types, including immune cell populations, pancreatic islets, platelets, hematopoietic stem and progenitor cells. In addition, we examine the expression profile of the constitutive proteasome and immunoproteasome across the different tissues and their correlation with HLA expression.

## Methods

### Datasets

**Fig. 1:**
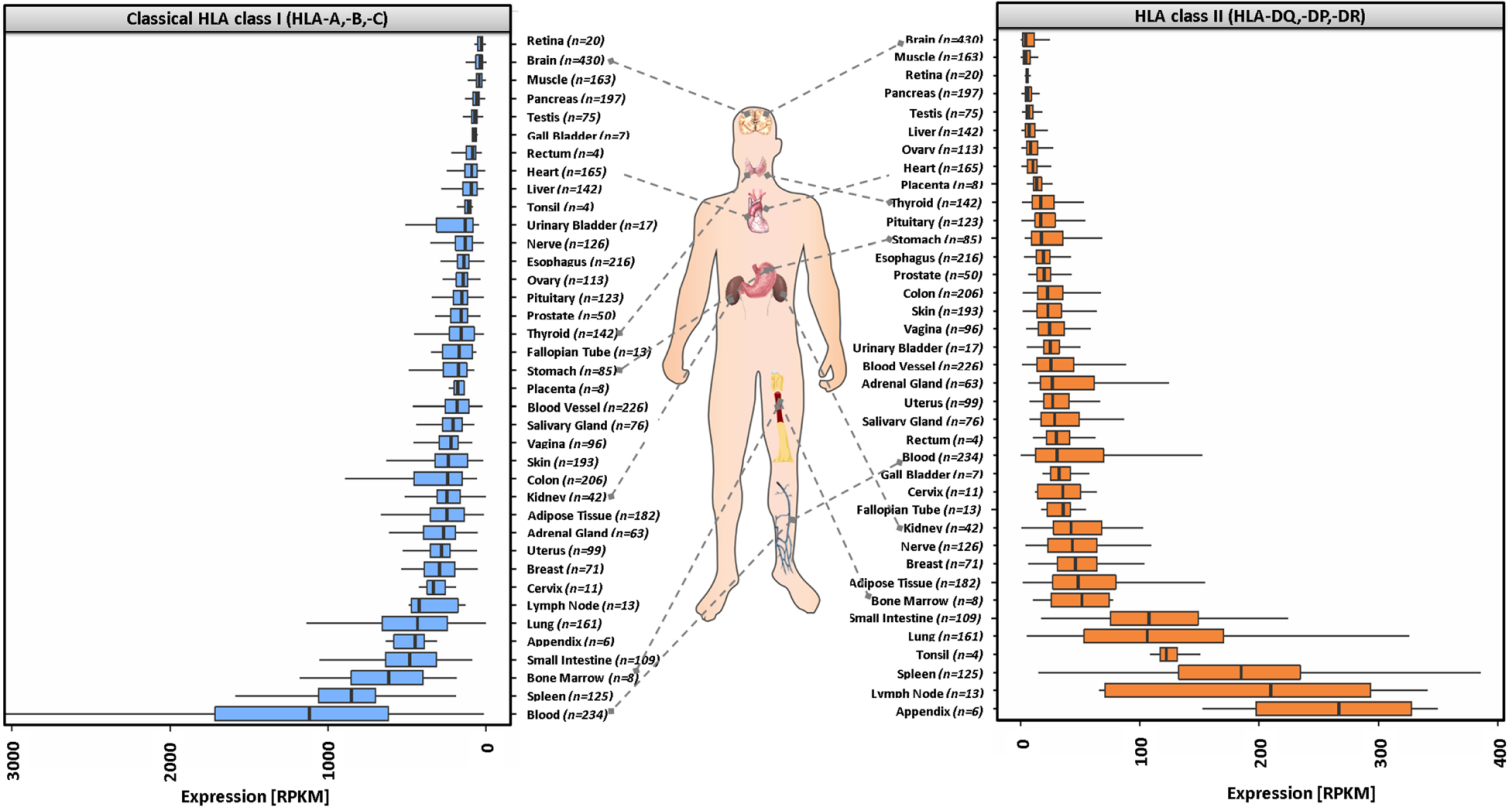
HLA class I and class II expression body map. NGS RNA-Seq data from human non-cancer tissues is re-analyzed using seq2HLA v2.4 to determine classical HLA class I (blue) and HLA class II (red) expression levels. Depicted are the median expression levels. Expression is normalized according *reads per kilobase of exon model per million mapped reads* (RPKM). Tissue images are derived from https://en.wikipedia.org/wiki/File:Fibromyalgia_symptoms.svg under “public domain” license

Paired-end RNA-Seq sequence reads for 4340 samples, representing 38 tissues and 17 cell types (Additional file 1: Table S1),sequenced with the Illumina platform were downloaded from the NCBI Sequence Read Archive (SRA) (http://www.ncbi.nlm.nih.gov/sra) and European Genome-phenome Archive (EGA) hosted by the European Bioinformatics Institute (EBI) (https://www.ebi.ac.uk/ega) [10] (Table 1). The number of biological replicates varies between 6 and 426 (appendix and brain, respectively) (Fig. 1). Sample etiology for the Genotype-Tissue Expression (GTEx) data is described at [http://www.gtexportal.org/home/tissueSummaryPage]: sample donors were primarily white males for whom the cause of death was anything from traumatic injuries (main cause of death for younger donors) to heart disease (main cause of death for over 60 year old donors).

**Table 1:** Downloaded datasets with Accession ID, Pubmed-ID and annotations, ^a^protected access

Dataset-Identifier	Study summary	Reference	# Samples	# Entities	Type	Comments
SRP012682^a^	Common Fund (CF) Genotype-Tissue Expression Project (GTEx); Protected access via dbGap (phs000424)	[16]	3926	30	tissues	non cancer
ERP003613, ERP006650	HPA RNA-seq normal tissues	[17]	204	30	tissues	non cancer
SRP017583	Transcriptome landscape of the human placenta	[18]	6	1	tissues	placenta
SRP051688	A cell-based systems biology assessment of human blood to monitor immune responses after influenza vaccination	[19]	55	7	cell types	immune cells
SRP056159	Disease-Associated SNPs from non-Coding Regions in Juvenile Idiopathic Arthritis Are Located Within or Adjacent to Functional Genomic Elements of Human Neutrophils and CD4+ T Cells	[20]	3	1	cell types	neutrophils
ERP003815	Next generation sequencing analysis of human platelet PolyA+ mRNAs and rRNA-depleted total RNA.	[22]	3	1	cell types	platelets
SRP010483	The human pancreatic islet transcriptome: expression of candidate genes for type 1 diabetes and the impact of pro-inflammatory cytokines	[21]	5	1	cell tyes	pancreatic islets
SRP034875	Comprehensive analysis of gene expression in human retina and supporting tissues	[58]	16	1	tissue	retina
SRP015336	Transcriptome analyses of the human retina identify unprecedented transcript diversity and 3.5 Mb of novel transcribed sequence via significant alternative splicing and novel genes.	[59]	4	1	tissue	retina
SRP058719	Long non-coding RNA profiling of human lymphoid progenitor cells reveals transcriptional divergence of B cell and T cell lineages.	[24]	18	5	cell types	hematopoietic stem cells
EGAD00001000745^a^	Transcriptional diversity during lineage commitment of human blood progenitors	[23]	83	8	cell types	hematopoietic stem cells

### HLA typing using RNA-Seq data

The tool seq2HLA v2.2 (https://github.com/TRON-Bioinformatics/seq2HLA) was used to derive HLA class I (classical and non-classical) and class II types from the RNA-Seq data. Seq2HLA v2.2 previously determined 4-digit resolution HLA types with high accuracy from classical HLA class I loci [9]. We extended seq2HLA v2.2 to automatically assign the 4-digit HLA types and determine the expression of the non-classical HLA class I genes by extending the HLA reference database with all known non-classical HLA allele sequences as described earlier [8]. HLA gene expression is normalized according to *reads per kilobase of exon model per million mapped reads* (RPKM) [6]. In addition, we extended seq2HLA v2.2 to not only detect the HLA class II molecules DQA1, DQB1 and DRB1, but also DRA, DPA1 and DPB1. As each locus (DP,DQ,DR) consist of the respective α- and β-chain, HLA class II expression is herein defined as the sum of min_rpkm(DPA1,DPB1), min_rpkm(DQA1,DQB1) and min_rpkm(DRA,DRB1). Seq2HLA v2.3 is freely available at https://github.com/TRON-Bioinformatics/seq2HLA.

### Gene expression analysis

Alignment of the raw reads against the humane reference genome build hg19 and calculation of gene-level expression of UCSC known genes is performed as described previously [11]. HLA gene expression is normalized according to *reads per kilobase of exon model per million mapped reads* (RPKM) [6]. To further validate the comparability of the RNA-Seq data [12], we additionally normalized the read counts according to *transcript per million* (TPM) [13] and reproduced the proteasome body map (Additional file 2: Figure S7, Additional file 1: Table S4). For TPM normalization, RPKM values for each sample are converted with: TPM = RPKM/(sum of RPKM over all genes) * 10^6.

### Statistical analysis

Correlation analysis is performed with R-3.1.2 for Linux. Throughout the study, the correlation coefficients are calculated according to Spearman’s rank correlation coefficient implemented in the package *psych*. Graphics are produced with the package *ggplot2* [14]. Differential expression analysis is performed with the R-package *DESeq2* [15] using the raw (i.e. not normalized values) read counts as input.

## Results

### Expression body map of classical HLA class I and HLA class II molecules

Human tissue RNA-Seq data was retrieved from the Genotype Tissue Expression consortium [16] (GTEx; 3926 samples from 30 tissues), the Human Protein Atlas [17] (204 samples from 30 tissues), as well as 20 retina samples from the Sequence Read Archive (Table 1, Additional file 1: Table S1). These samples were processed using seq2HLA [9] to obtain the HLA type as well as the expression levels of HLA class I (classical and non-classical) as well as HLA class II (Methods) and the results were integrated into a comprehensive HLA expression body map (Fig. 1, Additional file 2: Figure S1, Additional file 1: Table S2).

#### Classical HLA class I expression

Among the tissues with the highest median classical HLA class I expression (Fig. 1) are whole blood (1210 RPKM), spleen (850 RPKM) and bone marrow (615 RPKM). Interestingly, lung (433 RPKM) and small intestine (482 RPKM) showed similar median expression levels as the lymph node (422 RPKM). We observed the lowest median HLA class I expression in retina, brain and muscle tissues (29, 38 and 43 RPKM, respectively).

#### HLA class II expression

Overall, we found the median HLA class II expression levels across the different tissues (4 RPKM to 267 RPKM) to be much lower than the median classical HLA class I expression levels (29 RPKM to 1210 RPKM). Appendix (267 RPKM), lymph node (210 RPKM) and spleen (187 RPKM) showed the highest median HLA class II levels. Similar to classical HLA class I, we observed the lowest median HLA class II expression in brain (4 RPKM), muscle (4 RPKM) and retina (5 RPKM).

#### HLA expression in anatomical substructures

For a subset of tissues, GTEx and HPA provide additional annotation regarding the anatomical substructure of the sample (e.g. amygdala in brain tissue). Analysis of HLA expression in these substructures revealed very similar median classical HLA class I and HLA class II expression levels within the same tissue (Additional file 2: Figure S1). Of note, we found a clearly separated cluster of 9 samples (HLA class I expression larger than 900 RPKM) in brain, which are all derived from different brain regions (Additional file 2: Figure S1A) of a 63-year female donor (HLA class I and II types matches in all 9 samples). We examined the expression of immune-relevant genes (Methods, Additional file 2: Figure S2) in these brain samples and found the leukocyte marker CD45 enriched in the samples of the outlier group (median: 19.8 vs 2.6 RPKM), whereas the T-cell marker CD3E was very low or not expressed in both groups (median: 1.3 vs 0.1 RPKM). In contrast, the macrophage associated molecules CD68 (median: 236 vs 4 RPKM) and CSF1 (median: 47 vs 9 RPKM), the pathogen-associated molecular patterns sensing receptor TLR2 (median: 60 vs 2 RPKM), the transcription factor STAT1 (median: 549 vs. 36 RPKM), as well as the immune checkpoint molecule ligands PD-L1 (median: 41 vs. 1.3 RPKM) and PD-L2 (median: 11 vs. 0.3 RPKM) were clearly enriched in the outlier group. Finally, while the pro-inflammatory cytokine IFNγ and the tryptophan degrading enzyme IDO1 were not detectable in any of the samples with HLA class I expression below 400 RPKM (median: 0.03 and 0.02 RPKM), we found expression of these genes in the outlier group (median: 1.1 and 62.7 RPKM). Of note, the samples of this patient were also among the HLA class II high expressing outliers in brain (Additional file 2: Figure S1B).

### Expression of locus-specific HLA molecules

#### Classical HLA class I locus-specific expression

Examining the expression of the individual loci revealed a balanced classical HLA class I heavy chain expression within each tissue (Additional file 2: Figure S3A).

#### HLA class II locus-specific expression

α and β chains of the HLA class II loci HLA-DQ and HLA-DP are equally expressed, while HLA-DRA showed always slightly higher expression than HLA-DRB1. In all tissues, HLA-DR transcripts were always more abundant than HLA-DQ and HLA-DP transcripts (Additional file 2: Figure S3B).

### Expression body map of non-classical HLA class I molecules

We extended ‘seq2HLA’ to also determine the HLA allele and quantity of the non-classical HLA class I genes (i.e. HLA-E, -F, −G) and applied it to the RNA-Seq samples in order to examine their physiological expression (Additional file 1: Table S2).

#### HLA-G

**Fig. 2:**
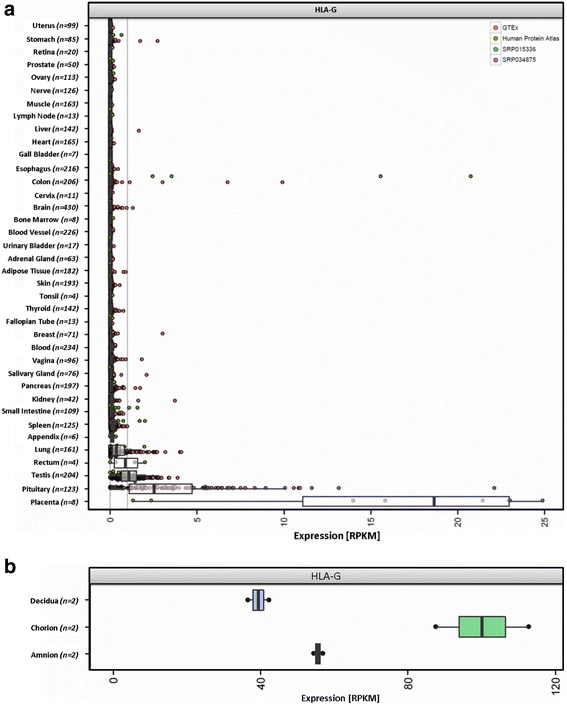
HLA-G expression body map. The non-classical HLA Class I gene HLA-G has immune tolerogenic functions, which is in contrast to the classical HLA molecules. **a** It shows a distinct pattern, with clear expression in placenta and pituitary gland, and to a lesser extent in testis, rectum and lung. **b** Confirmation of HLA-G expression in placenta from three different locations, with chorion showing the highest abundance of HLA-G transcripts

Analysis of HLA-G expression (Fig. 2a) showed a distinct pattern, with clear expression in placenta (median: 19 RPKM with 8 of 8 [100%] samples greater than 1 RPKM), pituitary (median: 2.5 RPKM with 96 of 123 [78%] samples greater than 1 RPKM) and testis (median: 1.1 RPKM with 113 of 204 [55%] samples greater than 1 RPKM). We confirmed the expression of HLA-G in placenta using RNA-Seq data from three different placental locations (amnion, chorion, decidua) produced in a different study [18] (Fig. 2b). Of note, the majority of lung samples showed a lack of HLA-G (median expression across the cohort: 0.35 RPKM). However, we observed 29 of 161 (18%) lung samples with an expression greater than 1 RPKM. Similarly, colon is showing eight striking outliers with HLA-G expression ranging between 1 and 22 RPKM. Although two rectum samples showed HLA-G abundance greater than 1 RPKM, the total number (*n* = 4) is too low to draw any conclusions.

#### HLA-E

We found HLA-E transcripts expressed in all tissues with retina (5.6 RPKM), testis (8.6 RPKM), muscle, brain (both 13 RPKM) and pancreas (17.7 RPKM) showing the lowest median abundance. In contrast, we found highest median expression in blood (275 RPKM), spleen (187 RPKM), lung (142 RPKM) and adipose tissue (141 RPKM) (Additional file 2: Figure S4A, Additional file 1: Table S2).

#### HLA-F

HLA-F showed a similar, but lower expression profile to that of HLA-E (Additional file 2: Figure S4B, Additional file 1: Table S2). Again retina (0.6 RPKM), muscle (2.8 RPKM), brain, pancreas (both 3.4 RPKM) and testis (4.8 RPKM) had the lowest expression, while spleen (119 RPKM) and whole blood (50 RPKM) showed the highest abundance of HLA-F transcripts.

### HLA expression in immune cell populations, platelets and pancreatic islets

**Fig. 3:**
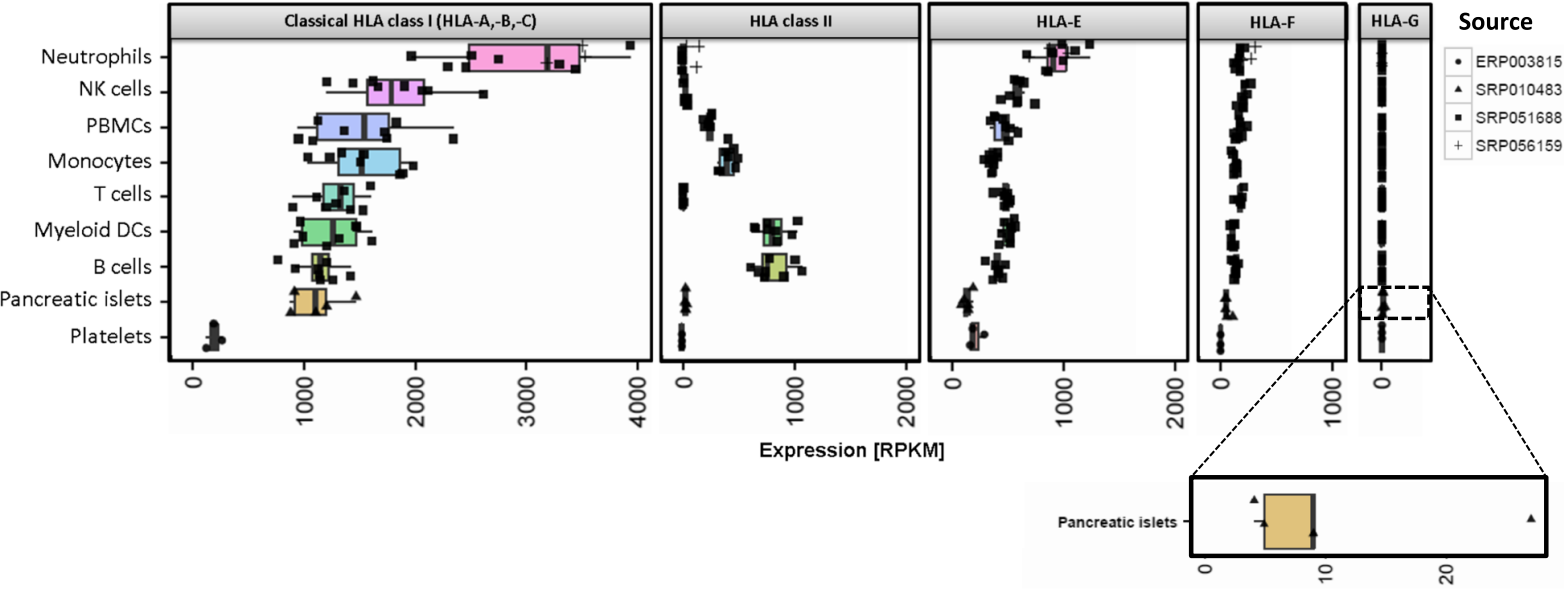
HLA expression in immune cell populations, platelets and pancreatic islets. Neutrophils show the highest and platelets the lowest classical HLA class I expression. HLA class II is most abundant in myeloid dendritic cells (myeolid DCs) and B cells. HLA-E – and to a lesser extent – HLA-F are ubiquitously expressed with the lowest abundance in pancreatic islets and platelets. HLA-G transcripts are found in pancreatic islets only

We analyzed RNA-Seq data of six different immune cell populations as well as peripheral blood monocytes (PBMCs) from volunteers prior to Influenza vaccination [19], as well as RNA-Seq data from neutrophils [20] (Fig. 3, Additional file 1: Table S5). High levels of classical HLA class I (median: 1148 RPKM – 3194 RPKM) were found in all immune cells, with neutrophils showing the highest median classical HLA class I and HLA-E expression (3194 RPKM and 843 RPKM respectively). The largest median HLA class II expression was observed in B cells (696 RPKM), myeloid dendritic cells (mDCs, 639 RPKM) and monocytes (323 RPKM), whereas HLA class II transcripts were almost absent in neutrophils (3 RPKM) and low abundant in T cells (10 RPKM). HLA-E (363–899 RPKM), and to a lesser extent HLA-F (110–210 RPKM) transcripts were ubiquitously found among all immune cell types, whereas HLA-G was not detected at all (0.05–0.5 RPKM). We also analyzed RNA-Seq data of human pancreatic islets [21] and found high classical HLA class I expression levels (median: 1111 RPKM), comparable with B cells, as well as much lower HLA class II transcript abundance (median: 38 RPKM) similar to NK cells. Again, we detected HLA-E and to a lesser extent HLA-F transcripts (140 RPKM and 55 RPKM). Of note, we found HLA-G to be expressed with a range of 5 RPKM and 27 RPKM (median: 9 RPKM) in pancreatic islets. Finally, we examined HLA expression in human platelets from four healthy blood donors [22]. Platelets showed the lowest classical HLA class I expression levels of all examined purified cell types (median: 192 RPKM), comparable amount of HLA-E (median: 182 RPKM), no HLA class II, no HLA-G and very low levels of HLA-F (median: 2.4 RPKM) transcripts.

### Human stem and progenitor cells

**Fig. 4:**
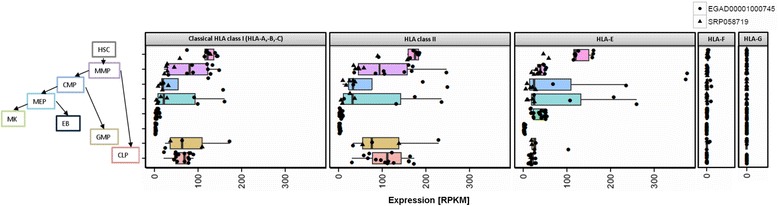
HLA expression in stem and progenitor cells. Expression profiles in HLA class I, class II and non-classical HLA class I transcripts in in hematopoietic progenitor cell populations (HSC=Hematopoietic Stem Cell, MMP = Multipotent Progenitor, CMP=Common Myeloid Progenitor, MEP = Megakaryocyte Erythrocyte Progenitor, MK=CD34- CD41+ CD42+ megakaryocytes, EB = Erythroblasts, GMP = Granulocyte Monocyte Progenitor, CLP=Common Lymphoid Progenitor)

Very recently, two different projects sequenced human blood progenitor cells [23, 24]. We analyzed and integrated both datasets to examine HLA expression during hematopoietic development (Fig. 4, Additional file 1: Table S6). Hematopoietic stem cells (HSC) showed comparable median classical HLA class I, class II and HLA-E levels (122, 177 and 120 RPKM, respectively). They differentiate into multipotent progenitor (MMP) cells, which showed lower median expression levels of classical HLA class I (81 RPKM), class II (96 RPKM) and much lower median abundance of HLA-E (40 RPKM). Common myeloid progenitor (CMP) cells showed low levels of HLA class I (median: 20 RPKM), HLA class II (median: 35 RPKM) and HLA-E (median: 20 RPKM). CMP cells differentiate into Megakaryocyte–erythroid progenitor (MEP), which showed a comparably low median HLA expression pattern (HLA class I: 22 RPKM, HLA class II: 33 RPKM, HLA-E: 26 RPKM). Megakaryocytes (MK) and erythroblasts (EB) showed even lower median HLA class I (6 and 3 RPKM, respectively) and HLA class II expression levels (5 and 3 RPKM respectively). While HLA-E is also very lowly expressed in EBs, we found higher expression levels in MKs (median: 41 RPKM), comparable to MMP cells. CMP cells can also differentiate into granulocyte monocyte progenitors (GMP), which showed a higher median classical HLA Class I (61 RPKM) and HLA class II (78 RPKM) expression profile, as well as similar HLA-E expression (median: 26 RPKM) compared to its progenitor. Finally, common lymphoid progenitor (CLP) cells showed similar median HLA expression profiles (classical HLA class I: 69 RPKM, HLA class II: 113 RPKM, HLA-E: 18 RPKM) compared to their progenitor cells (MMP). While ubiquitously abundant in all examined tissues and immune cells, HLA-F was only detected in HSCs and HLA-G was absent in all progenitor cells.

### Expression bodymap of constitutive proteasome and immunoproteasome

**Fig. 5:**
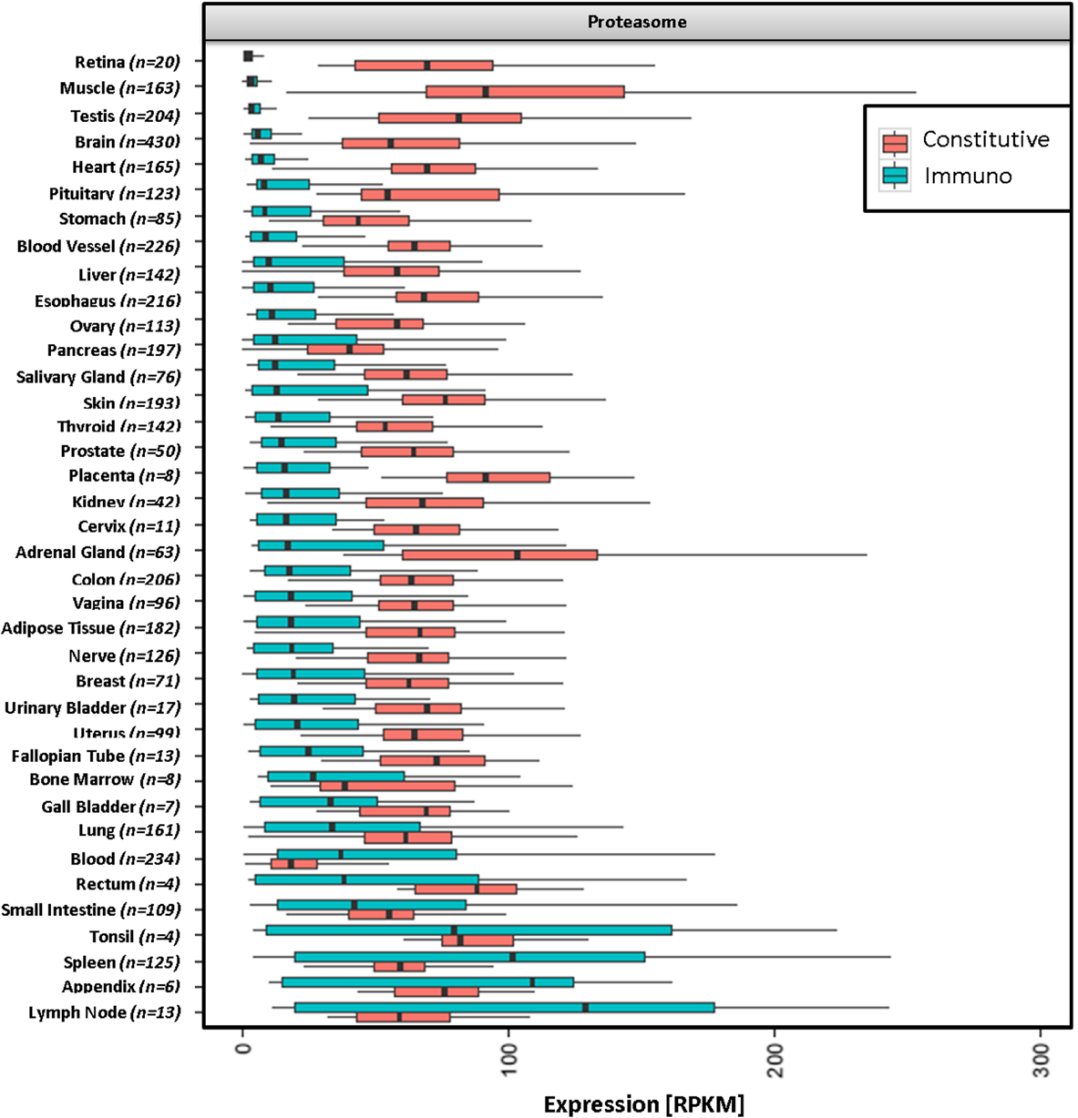
Expression of constitutive proteasome versus immunoproteasome. Median expression level of the genes PSMB5, PSMB6, PSMB7 from the constitutive proteasome (red) and median expression level of PSMB8 (LMP7), PSMB9 (LMP2), PSMB10 (LMP10) from the immunoproteasome (blue) are depicted to estimate expression of the respective proteasome type across different tissues. Sorted in ascending order of expression of the Immunoproteasome

In addition to the HLA class I (classical and non-classical) and HLA class II body map, we examined the expression profiles of proteasome components (PSMB5–10) across the 38 different tissues. The abundance of the constitutive proteasome is approximated by the median expression values of its three proteolytic subunits PSMB5, PSMB6 and PSMB7. Similarly, the immunoproteasome is represented by the median expression of the genes PSMB8 (LMP7), PSMB9 (LMP2), PSMB10 (LMP10). The constitutive proteasome was found in all tissues with blood (17 RPKM), bone marrow (38 RPKM) and pancreas (39 RPKM) having the lowest and placenta (91 RPKM), muscle (93 RPKM) and adrenal gland (103 RPKM) having the highest expression. In contrast, the immunoproteasome showed a higher variance. We detected very low expression of PSMB8 (LMP7), PSMB9 (LMP2) and PSMB10 (LMP10) in immune privileged tissues such as retina (2 RPKM), muscle (3 RPKM), testis (3.6 RPKM), whereas lymphoid tissues including spleen (101 RPKM), appendix (109 RPKM) and lymph node (129 RPKM) displayed highly abundant immunoproteasome levels (Fig. 5, Additional file 1: Table S3). These findings can be reproduced when normalizing the gene expression values according to *transcripts per million* (TPM, Additional file 2: Figure S7, Additional file 1: Table S4). Interestingly, expression profile of TAP1 and TAP2 resembles that of the immunoproteasome: retina (TAP1: 6.3 RPKM, TAP2: 5.9 RPKM), muscle (TAP1: 10.2 RPKM, TAP2: 7.2 RPKM) and brain (TAP1: 10.6 RPKM, TAP2: 10.1 RPKM) are amongst the tissues with the lowest amount of transcript abundance, whereas spleen (TAP1: 117.3 PRKM, TAP2: 60.2 RPKM), appendix (TAP1: 138.1 RPKM, TAP2: 98.8 RPKM) and lymph node (TAP1: 143 RPKM, TAP2: 112.2 RPKM) show the highest expression (Additional file 2: Figure S8A, Additional file 1: Table S8).

## Discussion

By analyzing 4340 public RNA-Seq datasets from 38 human tissues and 20 different hematopoietic cell populations, we generated a comprehensive expression atlas of classical (HLA-A, HLA-B, HLA-C) and non-classical (HLA-G,-E,-F) class I and class II (HLA-DPA1,-DPB1,-DQA1-DQB1,-DRA, -DRB1) HLA molecules.

### Classical HLA class I expression

We observed an extraordinary heterogeneity in median classical HLA class I expression levels between the tissues. Amongst the organs with the highest expression are spleen and bone marrow. They are key components of the lymphatic system accumulating immune cells, such as antigen presenting cells (APCs) in spleen and developing lymphocytes in bone marrow. The largest median abundance of classical HLA class I transcripts, however, can be found in whole blood. This might be explained by its cell type composition with neutrophils representing the largest population of nucleated cells (30–80%) [25]. We found neutrophils to have the highest classical HLA class I expression compared to other immune cells. Of note, we found classical HLA class I (and class II) transcripts highly abundant in lung and small intestine. This can be explained by the fact that the lung is exposed to the environment dealing first line with pathogens and that the small intestine contains lymphoid nodules (Peyer’s patches), which are part of the lymphatic system. In contrast, we observed the lowest classical HLA class I expression in brain, retina and testis, which are considered immune privileged [26–28] and muscle, which is described as an immunologically unique tissue [29]. Examining locus-specific expression, we found balanced expression of the individual classical HLA class I loci (HLA-A, -B, and –C) within each tissue. This result is in concordance with findings in human cell lines [30], but this is the first time that it has been established across a large collection of diverse human tissues. Although the density of HLA-C molecules on the cell surface has been reported to be much lower compared to HLA-A and HLA-B molecules [31, 32], we found that they are equally abundant on transcript level.

We also detected inter-individual variation of HLA expression within samples derived from the same organ. For example, we found that some brain samples showed a high-level transcription of HLA genes giving rise large variances in classical HLA class I expression (ranging between 0.4 RPKM and 1763 RPKM). To explore this in more detail we analyzed a group of outlier samples (*n* = 9) derived from different brain regions (Additional file 2: Figure S2) of the same patient showing an extremely high HLA class I expression > 900 RPKM. The samples were derived from post-mortem sections of a 63-year old woman who died of renal failure as consequence of an advanced chronic liver disease (hepatorenal syndrome, HS). Analysis of cell type specific markers in the RNA-Seq data of these samples showed highly abundant expression of CD45 and CD68 and upregulation of inflammatory markers such as interferon-γ (IFNγ) in agreement with a neuro-inflammation [33], probably mediated by the HS associated functional derangement in the blood-brain barrier.

### HLA class II expression

Across all tissues, we observed a lower HLA class II expression compared to classical HLA class I levels. Under physiological conditions HLA class II expression is limited to specialized cell types such as B cells, monocytes, macrophages and dendritic cells that constitutively express large amounts of HLA class II transcripts (Fig. 3). Accordingly, we detected the highest class II expression in lymphoid tissues, whereas immune-privileged organs retina and brain showed the lowest median HLA class II expression. We also found muscle to express low amounts of HLA class II transcripts, which is in concordance with earlier studies reporting HLA class II molecules on myoblasts [34] acting as antigen presenting cells [35].

Our study also confirmed an imbalanced HLA class II locus expression: HLA-DR transcripts were always expressed higher than HLA-DQ and HLA-DP. Each HLA class II molecule consists of the α- and the β-chain and we found balanced expression of these chains within each locus, with the notable exception of HLA-DR, for which we always found higher HLA-DRA abundance compared to HLA-DRB1.

### Expression of non-classical HLAs

In our study, we advanced seq2HLA [9] to determine the identity and expression of non-classical HLA class I molecules (i.e. HLA-E, -F, −G).

In contrast to classical HLA class I molecules, HLA-G is involved in mediating immune tolerance by inhibiting proliferation and cytotoxicity of immune-competent effector cells, such as monocytes, NK cells, or T cells [36]. Physiologic HLA-G expression has been found in placental cytotrophoblasts contributing to the maternal tolerance toward the fetus [5], in testicular tissue playing a significant role in human spermatogenesis [37], and in pancreatic islets inhibiting autoimmune damage during insulin exocytosis [38]. Indeed, we found a tissue-specific tissue distribution of HLA-G with expression only in those three body regions, additionally in pituitary gland (Fig. 2), which has not been reported before and in pancreatic islets (Fig. 3) Like pancreatic islets, the pituitary gland is part of the endocrine system secreting hormones, a process that produces potentially immunogenic targets that might trigger autoreactive effector cells. Of note, both structures showed higher median classical HLA class I expression compared to the organ they belong to: pituitary gland (154 RPKM) as part of the brain (35 RPKM) and pancreatic islets (1110 RPKM) are located in the pancreas (58 RPKM).

Together our similar findings in pancreatic islets and pituitary gland i) suggest similar mechanisms in counter-acting high classical HLA class I expression, ii) suggest the prevention of autoimmunity by downregulating effector functions of potential auto-reactive lymphocytes and iii) further strengthens the hypothesis that HLA-G expression of endocrine cells might be correlated to their secretory activity [38].

Furthermore, we observed presence of HLA-G transcripts (greater than 1 RPKM) in 29 of 161 (18%) lung samples. This is in concordance with findings, that HLA-G is expressed by lung macrophages and dendritic cells in 25% of patients with nonmalignant respiratory diseases [39]. In all other examined tissues and immune cell populations, we did not find evidence of HLA-G expression, with exception of striking outliers in colon. This highly tissue-specific expression profile and its immune-suppressive function makes HLA-G an interesting target, especially for cancer research [40].

HLA-E is the least polymorphic non-classical HLA class I gene and was first described to bind the activating and inhibitory NK cell receptors (namely CD94/NKG2C and CD94/NKG2A), suggesting a regulatory function to NK cells [41]. In contrast, we found HLA-E broadly expressed, which is in line with studies identifying HLA-E in a variety of cells and tissues on mRNA [42] and protein levels [43]. HLA-F is the least studied non-classical HLA class I gene. Like HLA-E, it is also thought to mediate important immunosuppressive functions [2], but unlike HLA-G, its significance in cancers is not yet established [44, 45]. HLA-F showed an expression profile similar to HLA-E with regard to tissue distribution, but lower in intensity. Of note, the expression patterns of both molecules resemble those of classical HLA molecules with immune privileged tissues showing the lowest and lymphatic organs the highest expression of classical HLA class I, class II, HLA-E and HLA-F transcripts, suggesting common regulatory elements. Indeed, transcription of HLA-E and HLA-F is induced by class II transactivator (CIITA) [36, 46], which was first described as the master regulator of HLA class II genes [47] and was also found to play a role in the regulation of HLA class I transcription [48]. In contrast, HLA-G is not regulated by CIITA, implying a different and unique transcriptional regulation amongst the non-classical HLA class I genes [46] resulting in the distinct, tissue-specific expression pattern. By analyzing correlations between CIITA and HLA expression across tissues, we can reproduce these findings (Additional file 2: Figure S6).

In immune cell populations, we found HLA-E to be expressed by B cells, T cells, NK cells, monocytes and mDCs, further corroborating previous findings [49–51]. We see the highest HLA-E expression in neutrophils, which also exhibit the highest classical HLA class I abundance, further supporting the transcriptional relationship between those molecules, even in individual cell types. HLA-F is also expressed ubiquitously in all examined immune cells. Interestingly, HLA-F has been recently found to be expressed on activated lymphocytes while being absent in resting lymphocytes [52].

### Stem and progenitor cells

Stem and progenitor cells regulate homeostasis e.g. of blood cells or tissue regeneration. Understanding stem cell differentiation is crucial for examining the underlying mechanisms of development, regeneration of damaged tissues and for the development of therapeutic approaches against various diseases. Thus, we analyzed RNA-Seq data from two different projects and found consistently, that hematopoietic stem cells (HSCs) express classical HLA class I and class II transcripts in equal amounts. This finding strengthens the important role of not only classical HLA class I, but also HLA class II type matching of donor and host in allogeneic HSC transplantations [53].

Together, these data show that HLA transcript expression decreases during hematopoietic stem cell differentiation following the myeloid and subsequently the megakaryocyte-erythroid lineages, which give rise to cells with no HLA molecules (in case of erythroblasts differentiating to erythrocytes) or very low amount of classical HLA class I (in case of platelets). In contrast, expression of HLA transcripts stays constant and is upregulated following the lineages resulting in immune cells.

### HLA and proteasome expression

The proteolytic activity of the constitutive proteasome is mediated through its components β1 (PSMB6), β2 (PSMB7) and β5 (PSMB5) that are characterized by different cleavage specificities. Upon stimulation with INFγ or TNFα, the immunoproteasome is generated by upregulating the inducible subunits β1i (PSMB9, LMP2), β2i (PSMB10, LMP10) and β5i (PSMB8, LMP7). While the constitutive proteasome is ubiquitously expressed in all tissues with no obvious relation to HLA expression, the median abundance of immunoproteasomal components strongly correlates with median HLA class II (*R* = 0.89) and classical HLA class I (*R* = 0.62) expression. This correlation suggests a common regulatory mechanism. Indeed, the immunoproteasome as well as classical HLA expression is induced upon stimulation with INFγ or TNFα and we found the median expression of both cytokines associated with the immunoproteasomal components (Additional file 2: Figure S7). Of note, PSMB9 (LMP2) shares a common bidirectional promotor with TAP1 (Transporter associated with Antigen Processing 1) suggesting a coordinate regulation [54]. Analysis of mRNA abundance of both genes within individuals across the different tissues reveals a strong correlation (*R* = 0.85), further corroborating the link between PSMB9 (LMP2) and TAP1 expression (Additional file 2: Figure S8B).

## Conclusion

This comprehensive expression body map provides a reference catalog of HLA expression profiles in normal human tissues and cell types. We found the classical HLA class I and HLA class II molecules highly expressed in lymphatic tissues (such as lymph node and spleen) and lowest in immune privileged organs (brain, retina, testis, muscle). In addition, we defined the expression body map of two types of proteasomes: the constitutive proteasome is expressed ubiquitously with no obvious correlation to classical HLA class I and class II expression, whereas the immunoproteasome – which produces classical HLA class I epitopes that are more efficient in activating cytotoxic T cells - is enriched in tissues of the lymphatic system and almost absent in immune privileged organs.

Further development of the tool ‘seq2HLA’ allowed us to determine non-classical HLA class I expression levels. While HLA-E and HLA-F showed similar expression profiles to those of classical HLA molecules, HLA-G is exclusively present in immunologically protected (placenta) and immune privileged sites (testis), as well as sites of the endocrine system (pituitary gland, pancreatic islets).

Our findings not only provide a resource of cell type specific HLA expression, but also highlight extremely variable expression of the basic components of antigen processing and presentation in different cell types and indicate that extremely low expression of classical HLA class I molecules together with lack of immunoproteasome components as well as upregulation of HLA-G may be of key relevance to maintain tolerance in immune privileged tissues.

We anticipate, that this resource might be helpful in cancer research as baseline when examining HLA dysregulation in tumor cells as part of their immune-escape mechanisms or choosing targets for cancer immunotherapy. Especially the highly tissue-restrictive physiological expression and its immune-suppressive functions makes HLA-G an interesting molecule in cancer research: as tool for the development of universal (HLA class I negative) T cells for adoptive T cell transfer [55], blockade of HLA-G expression and function on tumor cells may be a potential target for antitumor therapy [40] or as diagnostic and prognostic biomarker [56]. However, when using standard RNA-Seq data from a bulk tumor sample in conjunction with seq2HLA to study HLA expression by tumor cells specifically, attention must be given to the actual tumor content of the sample. Signals detected by seq2HLA (and any other gene expression quantification tools) are average values of the whole sample. One promising solution will be RNA-sequencing of single cells isolated from different tumor regions using laser capture microdissection [57].

## Additional files

Additional file 1: **Table S1.** List of tissues and cell types. **Table S2.** Median HLA expression values of the GTEx and HPA samples (for Fig. 1, Additional file 2: Figure S1). **Table S3.** Correlation analysis of HLA expression and APM components (normalized according to RPKM). **Table S4.** Correlation analysis of HLA expression and APM components (normalized according to TPM). **Table S5.** Median HLA expression of immune cell populations. **Table S6.** Median HLA expression in stem and progenitor cells. **Table S7.** Differential expression analysis of brain samples (high HLA vs. Low HLA expression) for Additional file 2: Figure S2. **Table S8.** Median TAP1 and TAP2 expression values of the GTEx and HPA samples (for Additional file 2: Figure S8) (XLSX 2820 kb)

Additional file 2: **Figure S1.** HLA Class I (A) and II (B) expression body map including anatomical substructures. **Figure S2**. Immunological characterization of brain samples. **Figure S3**. Locus specific expression of classical HLA class I (A) and class II (B) genes across all examined tissues. **Figure S4**. Expression body map of the non-classical HLA Class I genes. (A) HLA-E transcripts are ubiquitously expressed in all tissues. **Figure S5**. Expression of constitutive proteasome versus immunoproteasome normalized with TPM. **Figure S6**. Correlation analysis of CIITA and HLA expression. **Figure S7**. Expression of constitutive proteasome, Immunoproteasome and its inducing cytokines. **Figure S8**. Expression of TAP1, TAP2 and PSMB9. (PPTX 5223 kb)

## Abbreviations

CLP: Common lymphoid progenitor
CMP: Common myeloid progenitor
EB: Erythroblasts
EBI: European Bioinformatics Institute
FACS: Fluorescence-activated cell sorting
GMP: Granulocyte monocyte progenitor
GTEx: Genotype-Tissue Expression
HLA: Human leukocyte antigen
HSC: Hematopoietic stem cell
IHC: Immunohistochemistry
MEP: Megakaryocyte erythrocyte progenitor
MHC: Major histocompatibility complex
MK: CD34- CD41+ CD42+ megakaryocytes
MMP: Multipotent progenitor
NCBI: National Center for Biotechnology Information
NGS: Next generation sequencing
RPKM: Reads per kilobase of exon per million mapped reads
RT-qPCR: Real-time quantitative polymerase chain reaction
SRA: Sequence read archive
TPM: Transcripts per million
