# Supplementary material for: HLA and proteasome expression body map
Source: BMC Med Genomics. 2018 Mar 27;11:36. doi: 10.1186/s12920-018-0354-x (PMC5872580; doi:10.1186/s12920-018-0354-x)
Supplement: Supplementary file 2 — Figure S1. HLA Class I (A) and II (B) expression body map including anatomical substructures. Figure S2. Immunological characterization of brain samples. Figure S3. Locus specific expression of classical HLA class I (A) and class II (B) genes across all examined tissues. Figure S4. Expression body map of the non-classical HLA Class I genes. (A) HLA-E transcripts are ubiquitously expressed in all tissues. Figure S5. Expression of constitutive proteasome versus immunoproteasome normalized with TPM. Figure S6. Correlation analysis of CIITA and HLA expression. Figure S7. Expression of constitutive proteasome, Immunoproteasome and its inducing cytokines. Figure S8. Expression of TAP1, TAP2 and PSMB9. (PPTX 5223 kb) [file 12920_2018_354_MOESM2_ESM.pptx]

## Slide 1
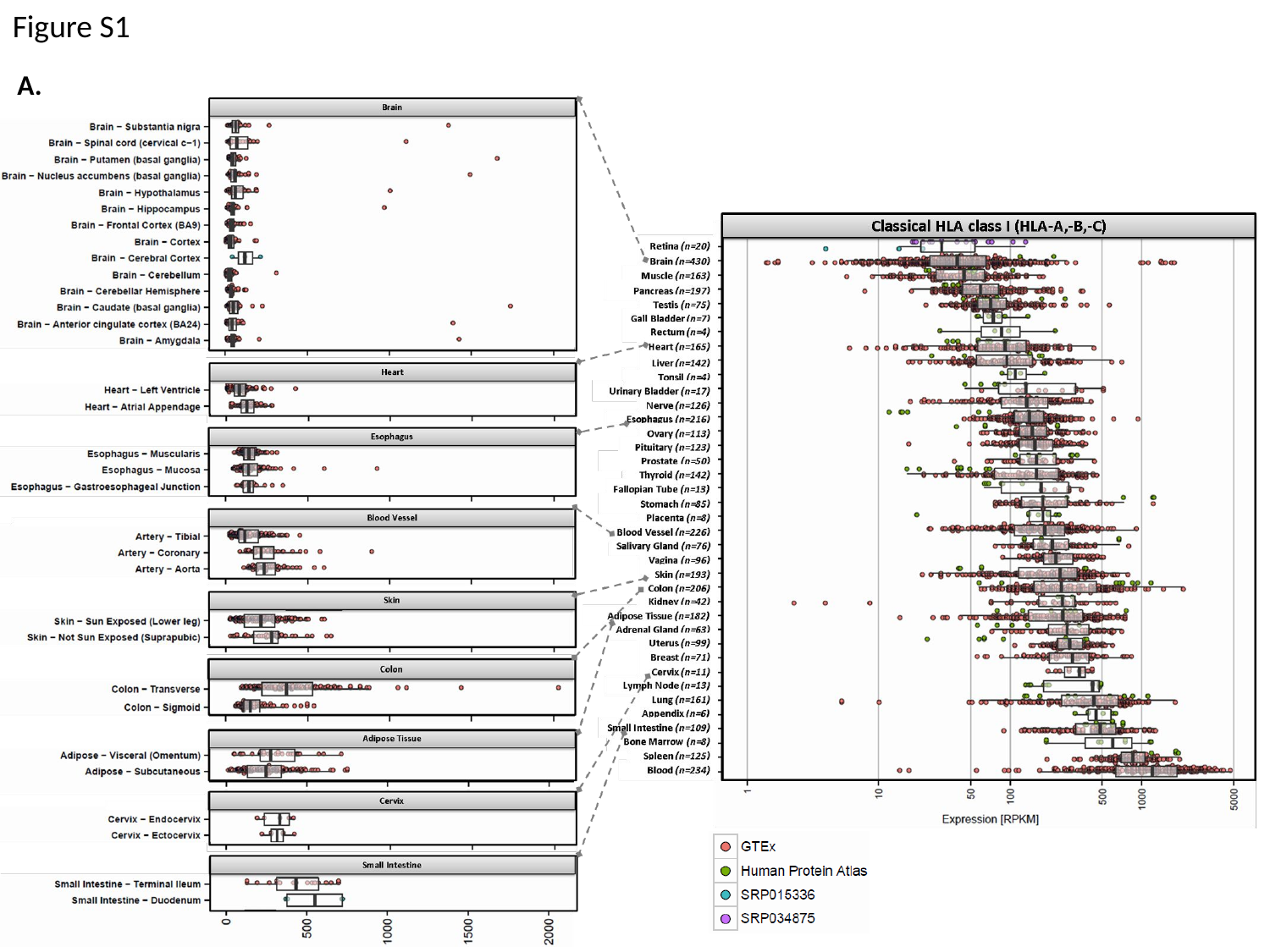

Figure S1
A.

## Slide 2
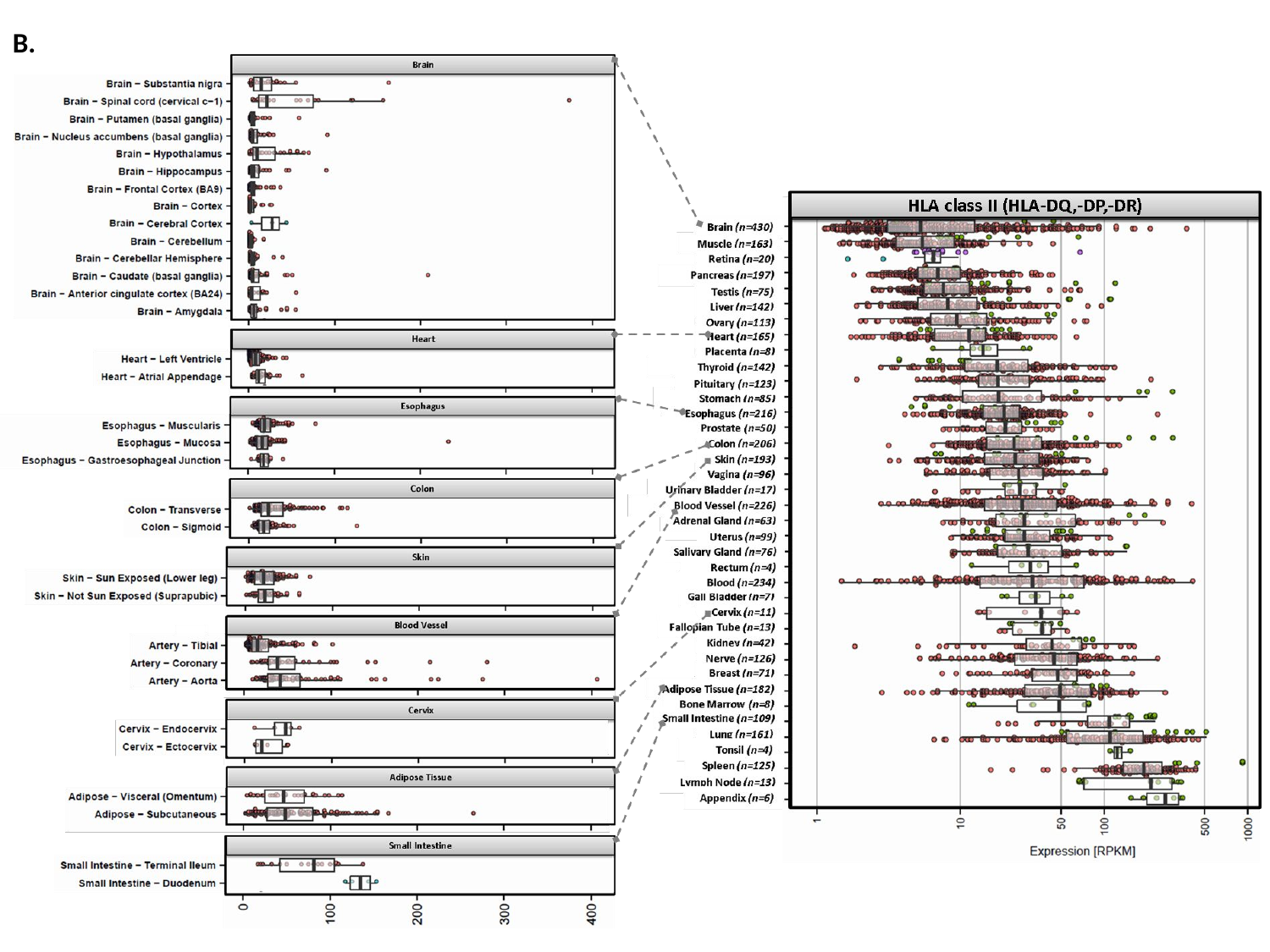

B.

## Slide 3
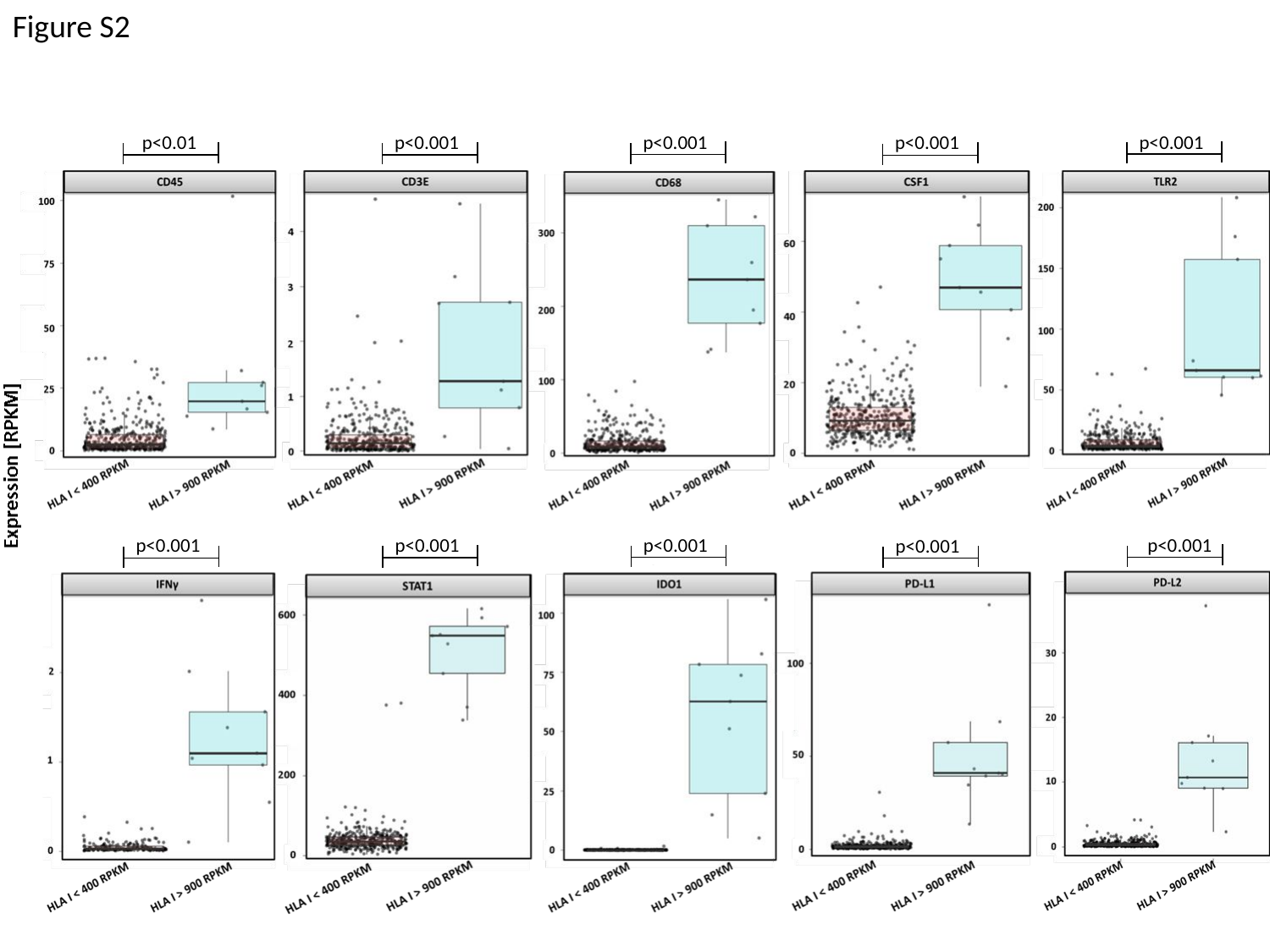

Figure S2
p<0.001
p<0.001
p<0.01
p<0.001
p<0.001
p<0.001
p<0.001
p<0.001
p<0.001
p<0.001

## Slide 4
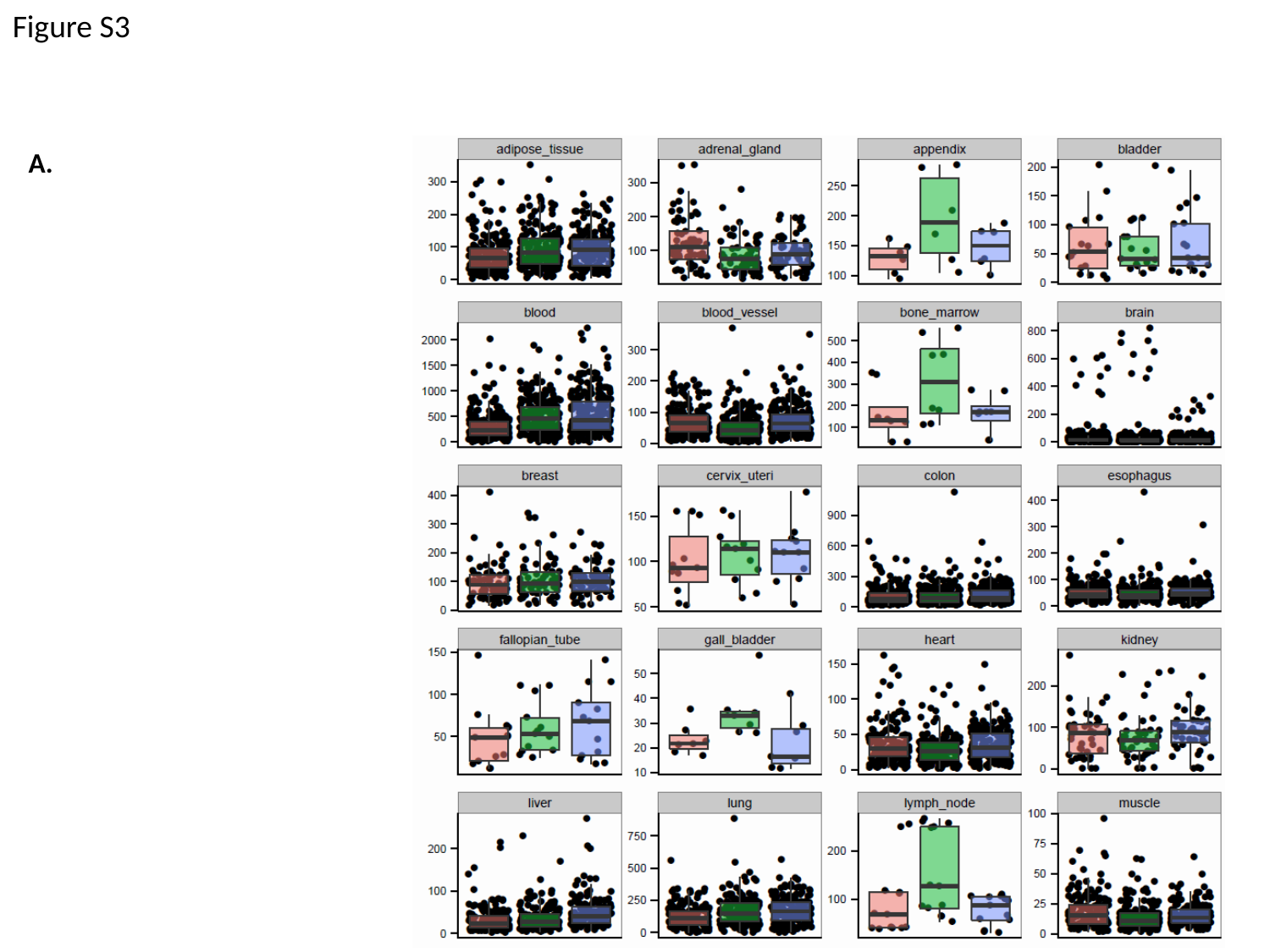

Figure S3
A.

## Slide 5
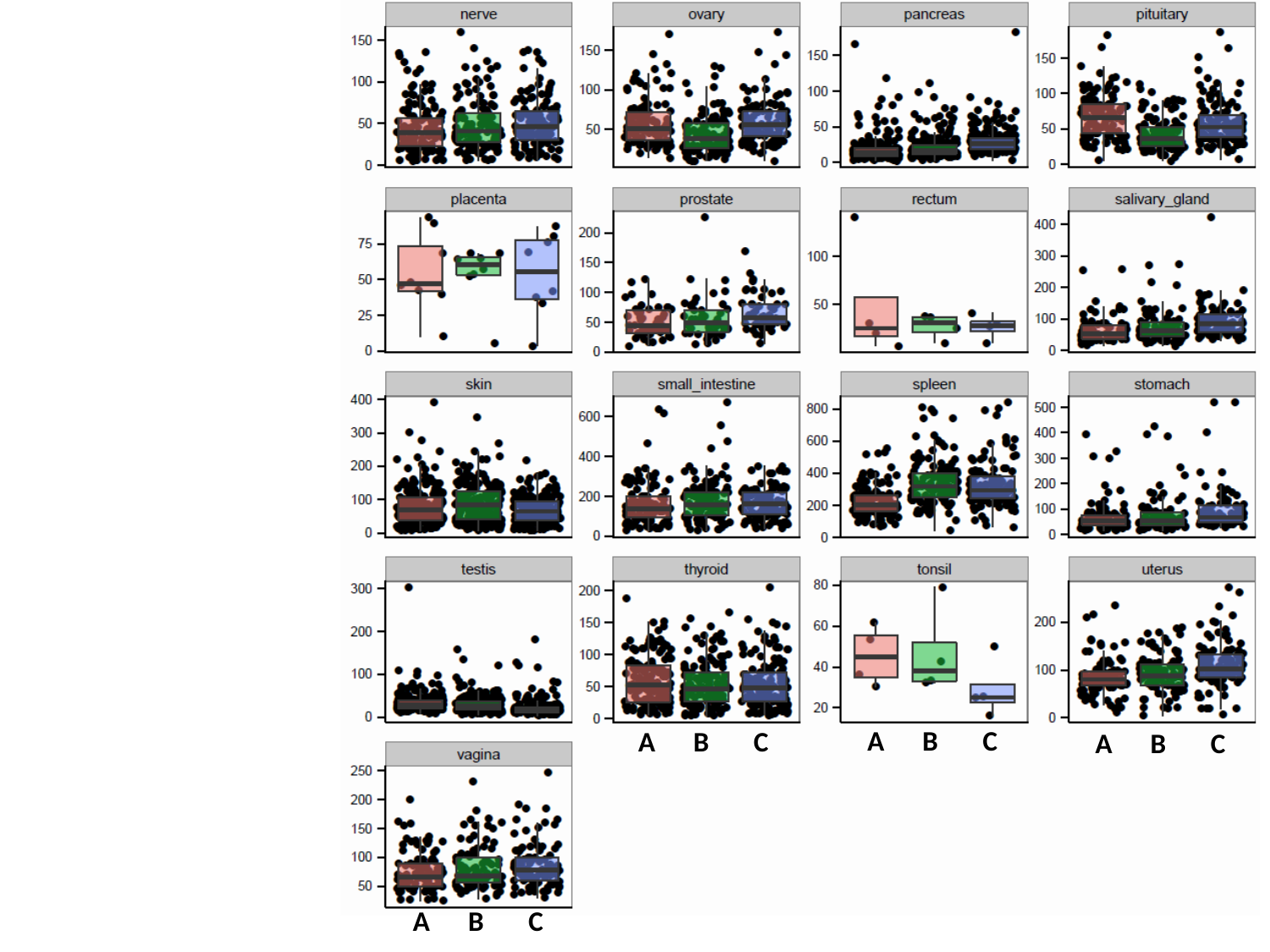

A B C
A B C
A B C
A B C

## Slide 6
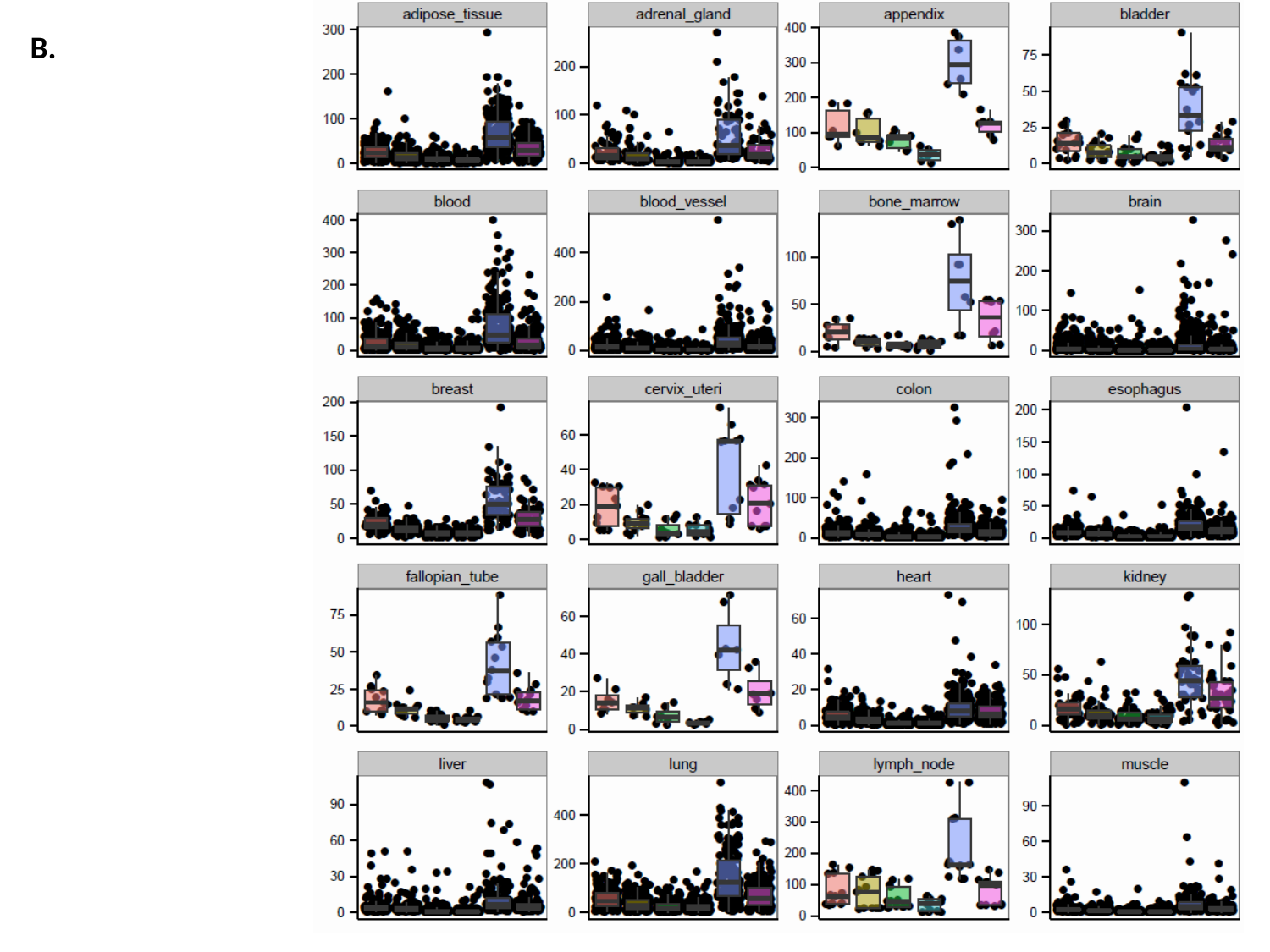

B.

## Slide 7
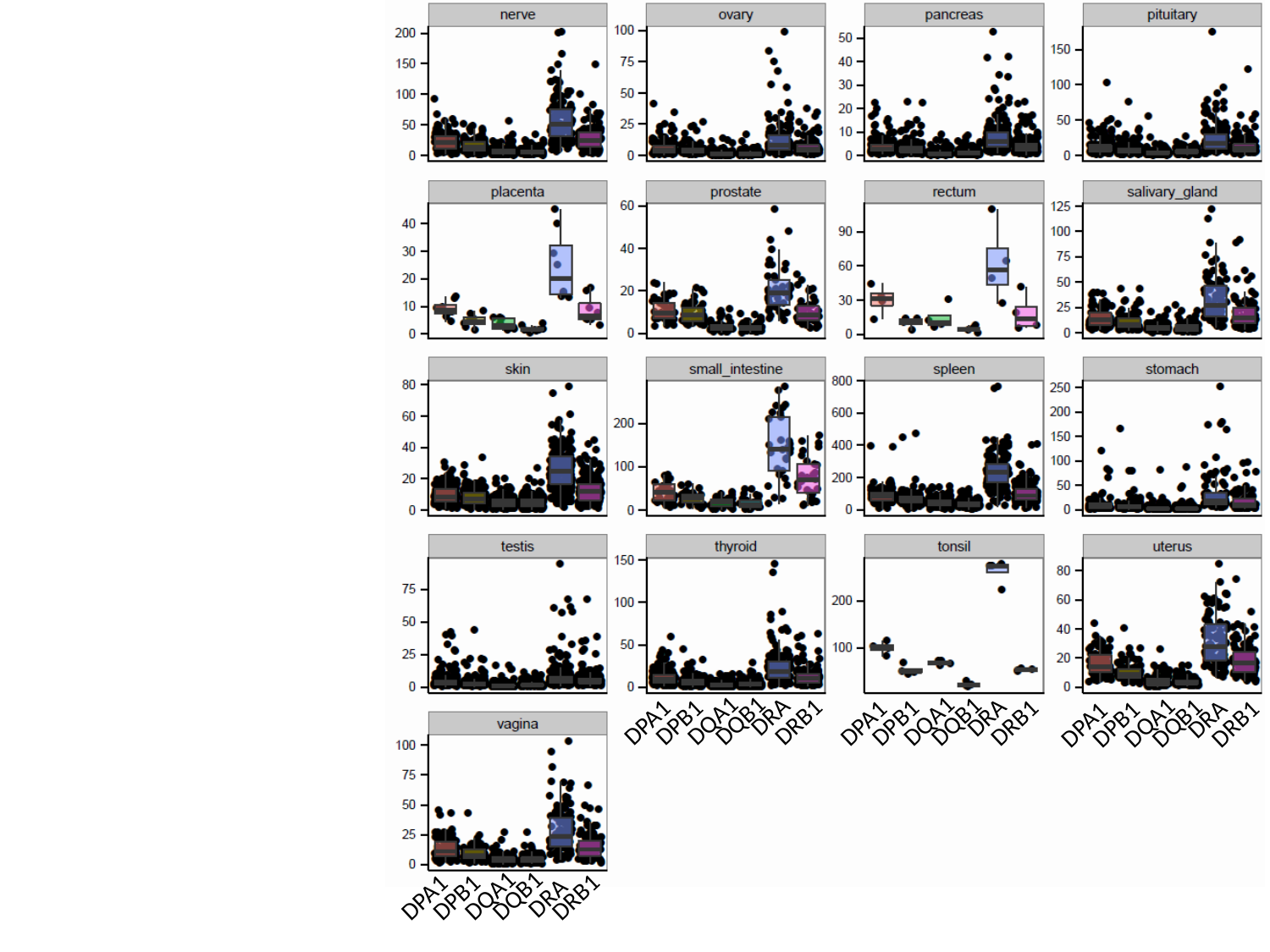

DRA
DRA
DRA
DPA1
DPB1
DQA1
DQB1
DRB1
DPA1
DPB1
DQA1
DQB1
DRB1
DPA1
DPB1
DQA1
DQB1
DRB1
DRA
DPA1
DPB1
DQA1
DQB1
DRB1

## Slide 8
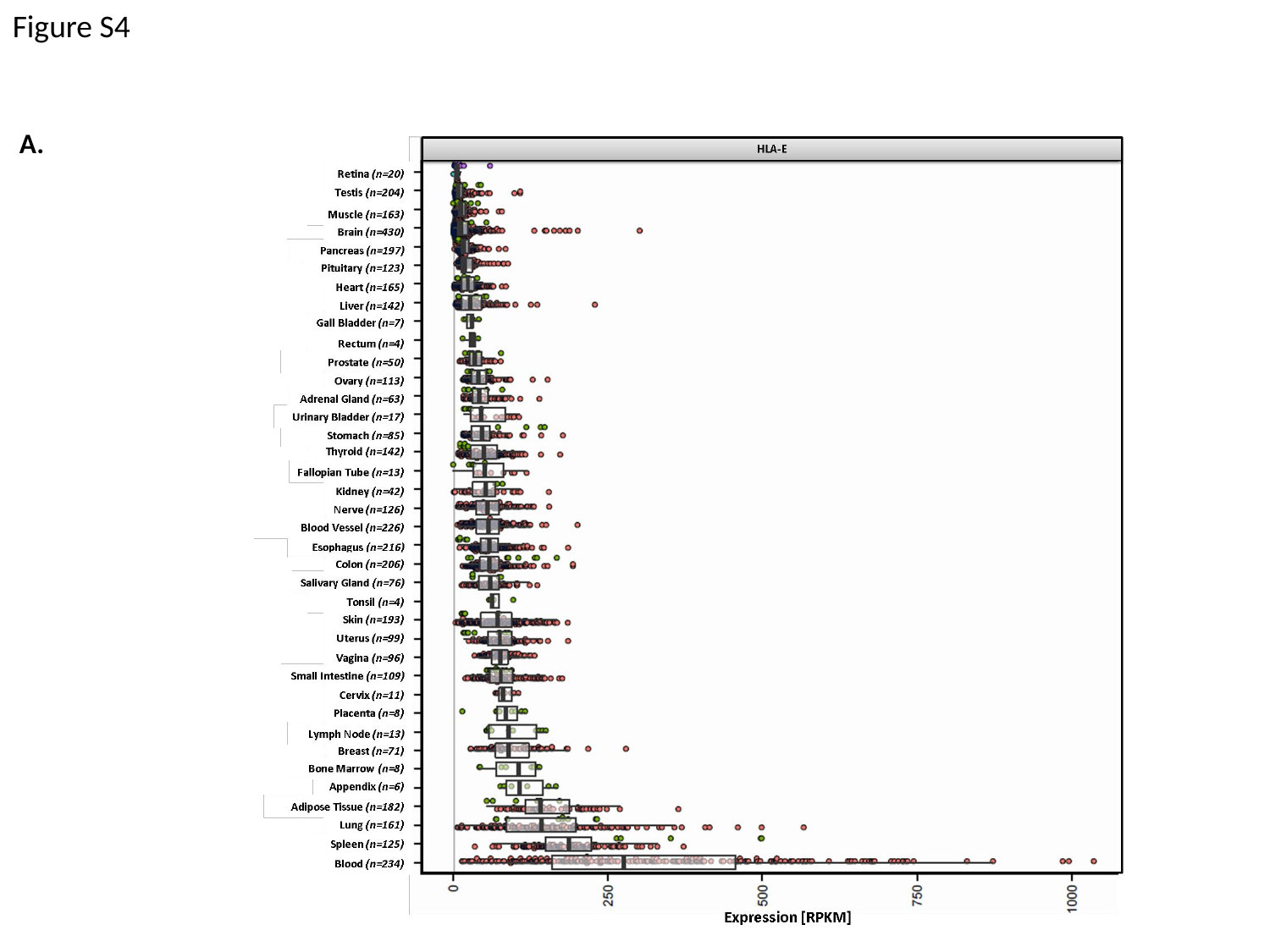

Figure S4
A.

## Slide 9
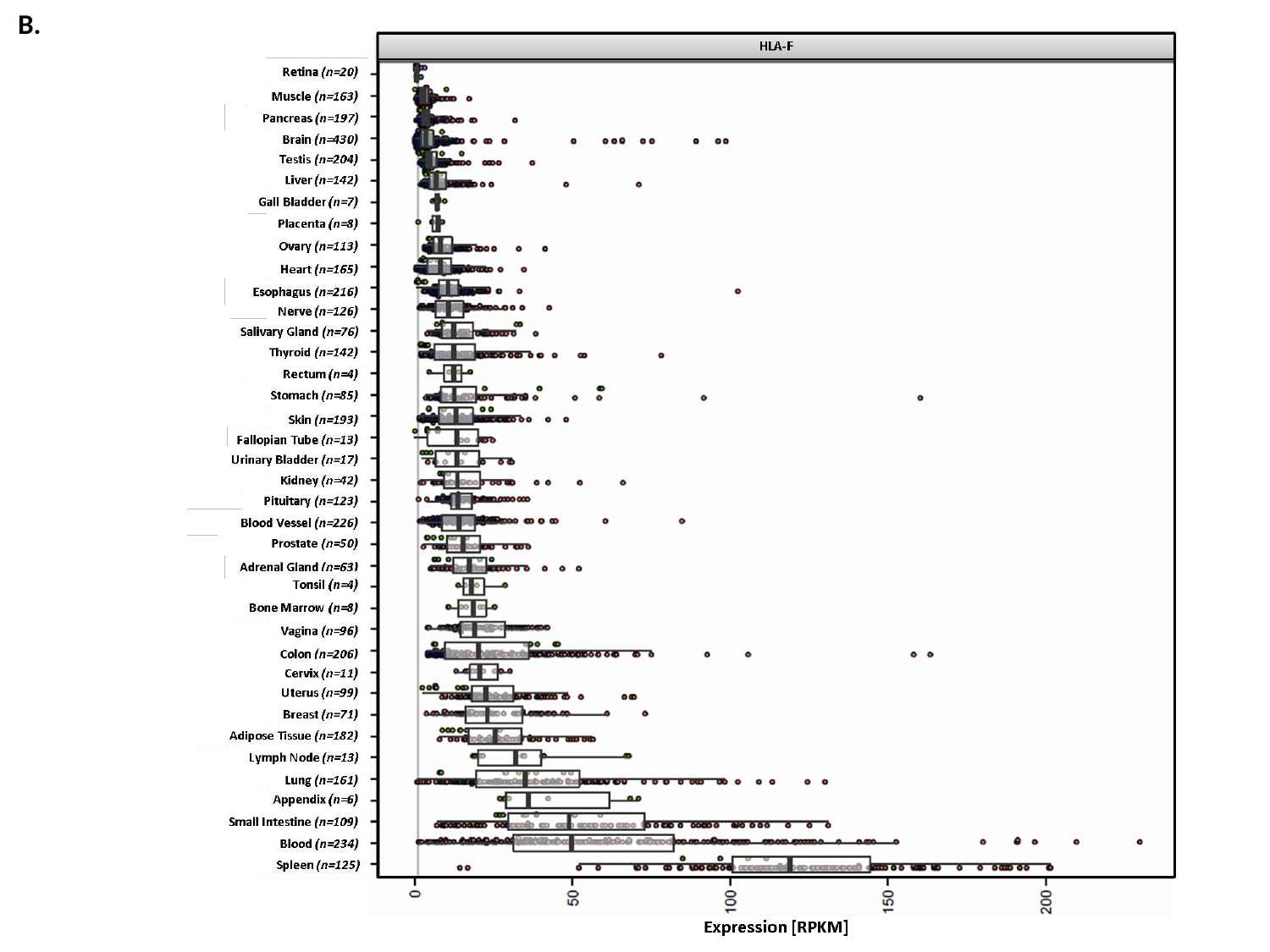

B.

## Slide 10
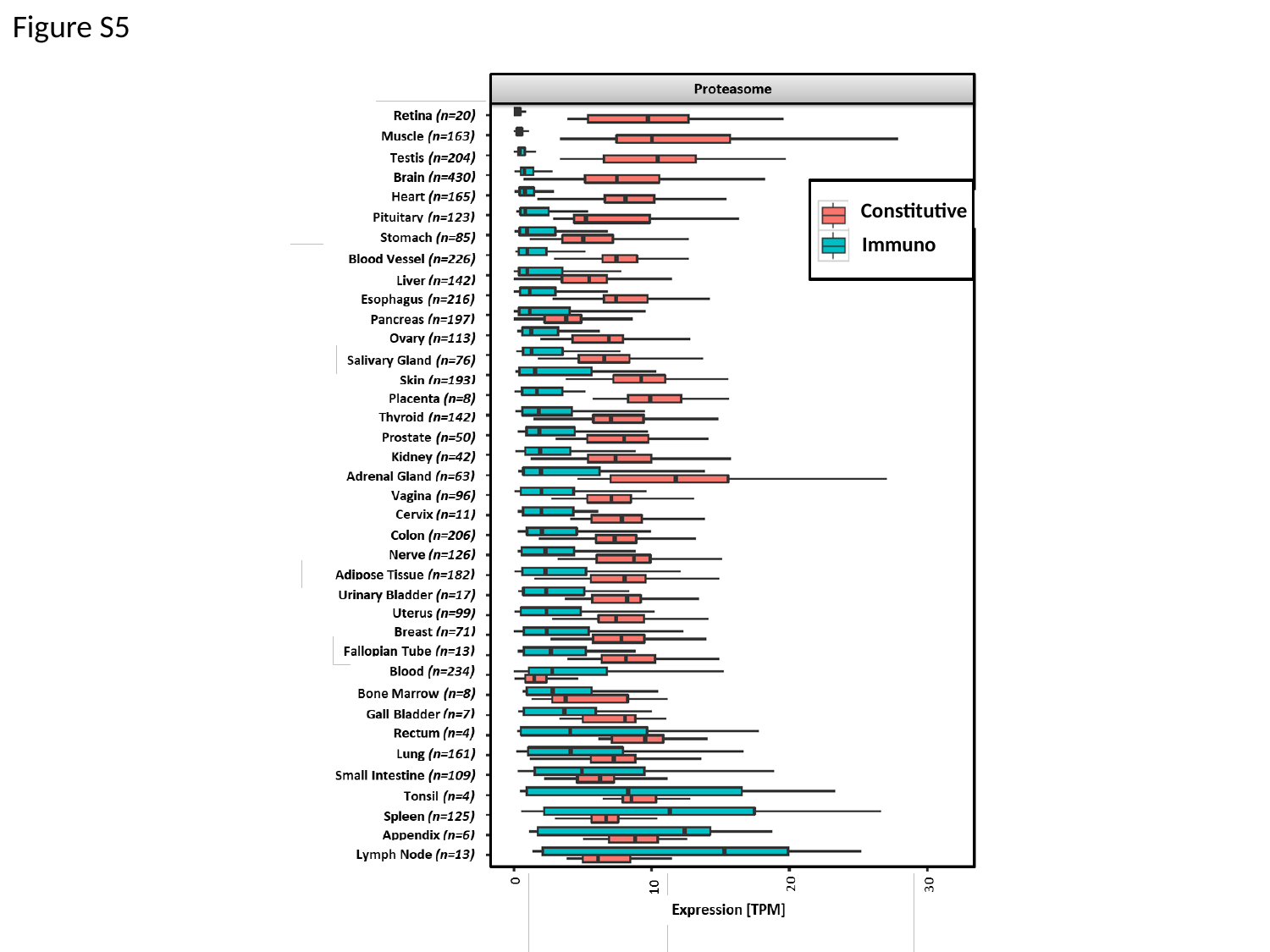

Figure S5
Constitutive
Immuno

## Slide 11
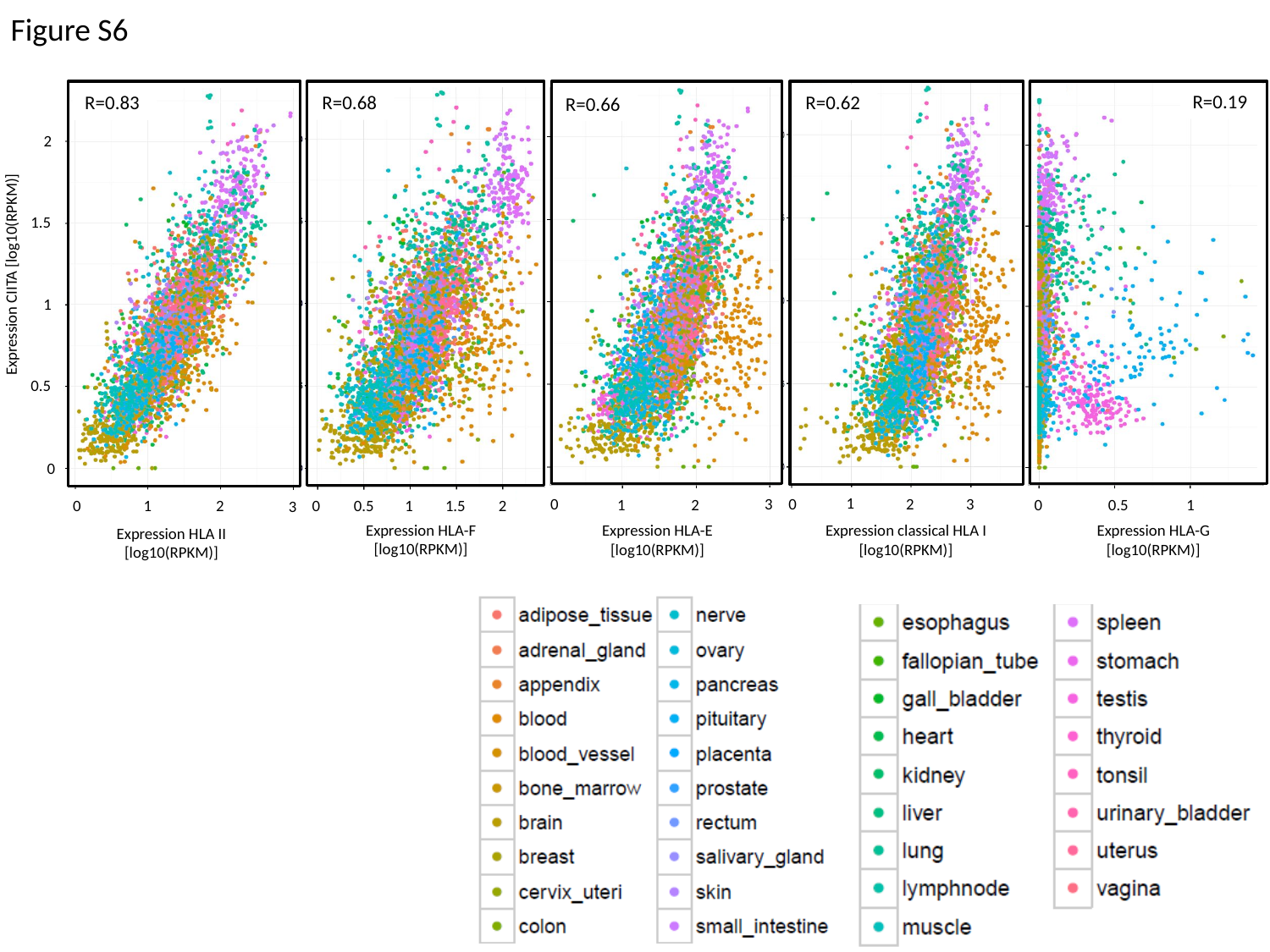

Figure S6
R=0.19
R=0.68
R=0.83
R=0.62
R=0.66
2
1.5
Expression CIITA [log10(RPKM)]
1
0.5
0
1
2
3
3
0
0
2
1
1
0
0.5
0
1
1.5
2
1
2
0
0.5
3
Expression HLA-F [log10(RPKM)]
Expression HLA-E [log10(RPKM)]
Expression classical HLA I [log10(RPKM)]
Expression HLA-G [log10(RPKM)]
Expression HLA II [log10(RPKM)]

## Slide 12
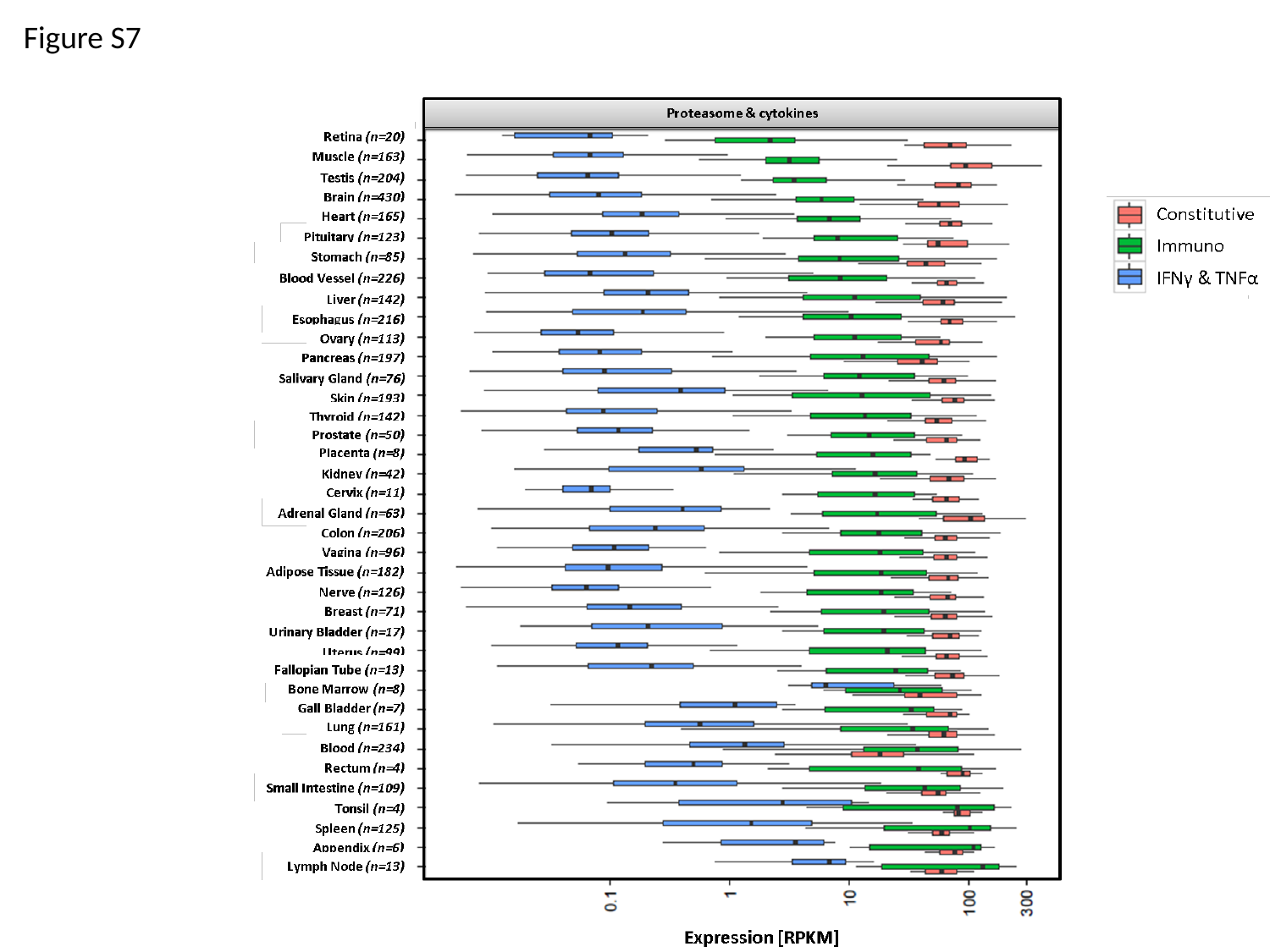

Figure S7

## Slide 13
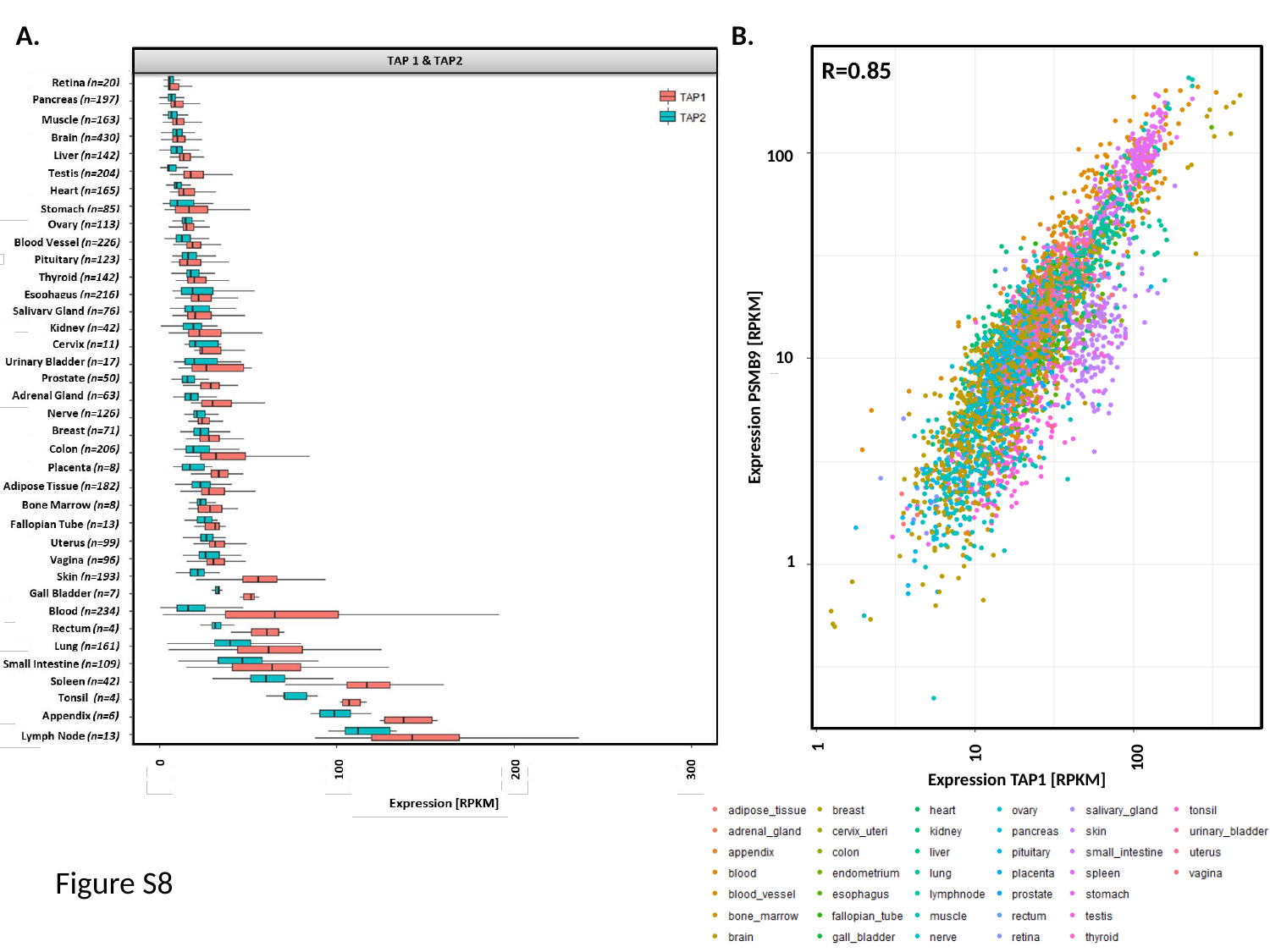

A.
B.
R=0.85
100
10
Expression PSMB9 [RPKM]
1
1
100
10
Expression TAP1 [RPKM]
Figure S8
